# Functional and Symptomatic Improvements Based on the Femoral Tunnel Drilling Technique in Anterior Cruciate Ligament (ACL) Reconstruction

**DOI:** 10.7759/cureus.65741

**Published:** 2024-07-30

**Authors:** Sarah C Kurkowski, Michael J Thimmesch, Sophia Le, Henry Kuechly, Lynessa McGee, Michael Kloby, Paul McMillan, Logan P Lake, Barton Branam, Christopher Utz, Brian Grawe

**Affiliations:** 1 Department of Orthopaedic Surgery, University of Cincinnati College of Medicine, Cincinnati, USA; 2 Medical School, Medical College of Wisconsin, Milwaukee, USA

**Keywords:** anteromedial portal, transtibial, rigid reaming, flexible reaming, femoral tunnel drilling, acl, knee ligaments

## Abstract

Background: The current literature comparing femoral tunnel techniques often reports on short-term outcomes after anterior cruciate ligament reconstruction (ACLR), but only a few studies have analyzed long-term outcomes. In addition, many studies have compared transtibial to anteromedial portal techniques without differentiating whether rigid or flexible reaming is used, making it difficult to infer how the techniques truly compare to one another.

Purpose: This study aimed to detect differences in patient-reported outcome scores in those treated with three different femoral tunnel drilling techniques.

Study design: This study is a prospective cohort study.

Methods: Of 650 patients treated for anterior cruciate ligament (ACL) injuries with ACLR, 350 were 5+ years out from surgery. Of these patients, 111 completed patient-reported outcome surveys (PROs). The Kruskal-Wallis H test was used to detect differences between patients treated with either of the three femoral tunnel drilling techniques: transtibial (TT), anteromedial portal with rigid reaming (AMP-RR), or anteromedial portal with flexible reaming (AMP-FR). Bonferroni correction was applied to the p-values to reduce the risk of making a type 1 error.

Results: No differences were found between the three groups in demographics or postoperative PROs. However, there was a significant change between pre-surgery and post-surgery PROs. TT, when compared to AMP-RR, had a greater increase in satisfaction and greater improvement in a patient’s ability to go up and down the stairs from pre-surgery to post-surgery. AMP-FR, when compared to TT, had greater improvement of the patient’s knee stiffness/swelling. AMP-FR, when compared to AMP-RR, had greater improvement in knee pain during stairs and the ability to go down the stairs. No differences in return to sport, additional procedures on the affected knee (meniscal surgeries or cyclops lesion excisions), or revision surgery rates were found.

Conclusion: Overall, postoperative PROs did not show statistically significant differences between the three femoral tunnel drilling techniques. Differences, however, were identified in the responses to specific questions on PRO surveys, which may have otherwise been overlooked. It is important to recognize the differences between TT, AMP-RR, and AMP-FR in the improvement of stair climbing and swelling/stiffness as these likely directly affect a patient’s satisfaction from pre-ACLR to post-ACLR.

## Introduction

Anterior cruciate ligament reconstruction (ACLR) has been the long-time standard of care for ACL insufficiency. An important part of the reconstruction is the appropriate placement and creation of the femoral tunnel where the graft is inserted, ideally mimicking the anatomic ACL insertion as closely as possible. However, the literature has found that anywhere from 10% to 40% of ACL bone tunnels are malpositioned during reconstruction. This ultimately predisposes the patient to worse outcomes as a reconstructed femoral tunnel outside of the normal anatomic placement puts the knee at risk for decreased stability after surgery. In addition, improper placement can cause impingement of the graft with the posterior cruciate ligament or the intercondylar notch or lead to abnormal tension on the ACL graft [1].

There are multiple techniques used by orthopedic surgeons to reconstruct the femoral tunnel during ACLR. These include over-the-top, outside-in, transtibial (TT), anteromedial portal with rigid reaming (AMP-RR), anteromedial portal with flexible reaming (AMP-FR), and retrograde reaming. The TT technique is dependent on the placement of the tibial tunnel; therefore, it has been criticized for placing the femoral tunnel too anterior and proximal to the anatomic ACL femoral footprint possibly leading to graft impingement and knee instability [1,2]. AMP-RR and AMP-FR techniques are independent of the tibial tunnel. AMP-RR allows for fewer incisions and closely mimics the ACL footprint. However, disadvantages include increased risk for posterior wall blowout, short femoral tunnel length, or damage to lateral knee structures. AMP-FR has similar advantages as AMP-RR such as fewer incisions, but the flexible reaming allows for anatomic positioning of the femoral tunnel with less risk of posterior wall blowout or damage to lateral structures [1,3-6].

Multiple studies in the current literature have examined TT in comparison to AMP-RR or AMP-RR to AMP-FR in both prospective and retrospective ways [6-10]. Literature reviews and meta-analyses have also attempted to compile these studies to gain a better understanding of which technique is best for femoral tunnel drilling. However, very few if any studies have simultaneously compared TT, AMP-RR, and AMP-FR, and many studies on this topic do not have mid-term or long-term follow-up on patients.

The purpose of this study was to prospectively compare three femoral tunnel drilling techniques (TT, AMP-RR, and AMP-FR) in ACLR. It was hypothesized that there would be no difference in patient-reported outcomes (PROs) from pre-surgery to 5+ years post-surgery in patients treated with one of these three femoral tunnel drilling techniques in ACLR.

## Materials and methods

Patient enrollment and data collection

This is a prospective study comparing three femoral tunnel drilling techniques (TT, AMP-RR, and AMP-FR) between patients undergoing ACL reconstruction. A total of 650 patients were prospectively enrolled starting in 2014 in the Department of Orthopaedic Surgery, University of Cincinnati, a single-center institutional ACL registry in Ohio, USA. Four sports medicine fellowship-trained orthopedic surgeons cared for all 650 patients at a tertiary academic medical center. Study approval was obtained from the University of Cincinnati Institutional Review Board (approval no. 2020-0116).

Demographic, preoperative, and intraoperative data were collected prospectively as the patient was seen for initial and follow-up visits related to their ACL injury and reconstruction. Demographic data included age, sex, body mass index (BMI), education level (reported as the highest grade or degree earned), health insurance type, and smoking status. Preoperative data included sports involvement, recreational activities, physical exam at the time of initial evaluation, and thigh circumference. Intraoperative data included femoral tunnel drilling technique, knee flexion during tunnel drilling, graft choice, size of graft, graft fixation, and concomitant knee injuries. One surgeon used the TT technique for the placement of the femoral tunnel, one surgeon used the AMP-RR technique, and two surgeons used the AMP-FR technique.

**Figure 1:**
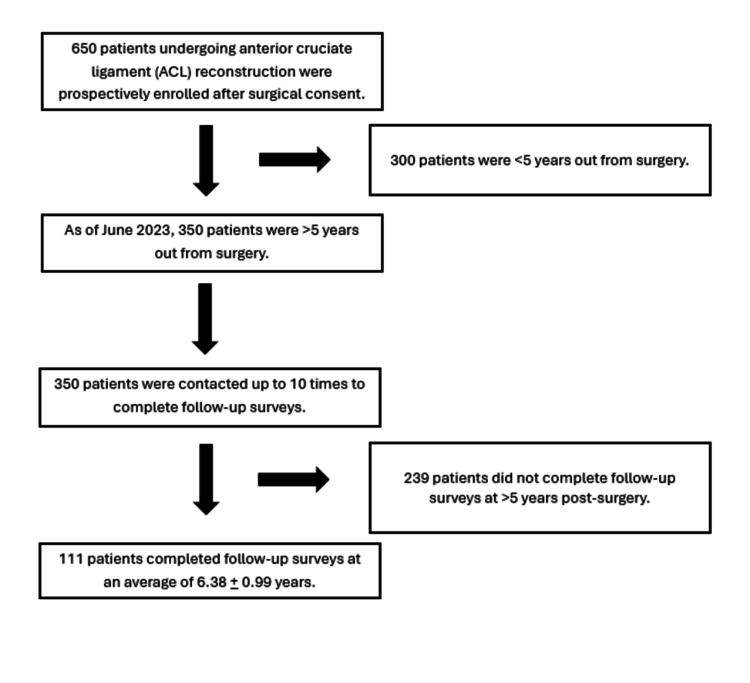
Patient recruitment and study design.

At each visit, patient-reported outcomes were collected and recorded in REDCap (Research Electronic Data Capture, Vanderbilt University, United States). The PROs included the International Knee Documentation Committee subjective evaluation form (IKDC) [11-12], Knee Injury and Osteoarthritis Outcome Score (KOOS) [13-14], Marx score [15-16], return to sport (RTS) after injury score, pain, and satisfaction [17-18]. The KOOS total score was broken down into its five sub-scores: symptom and stiffness, pain, function of daily living, function in sports and recreation, and quality of life. Pain was measured on a scale of 1 to 5, with 1 being “no pain” and 5 being “extreme pain." Satisfaction was measured on a scale of 1 to 5, with 1 being “very unsatisfied” and 5 being “very satisfied." Of the 650 patients, 350 were five or more years out from their ACL reconstruction. In addition, the change in PROs from preoperative to postoperative time points was calculated as well to gain insight into how each technique affected patients’ improvement after ACLR. Our team attempted to contact each of the 350 patients for follow-up surveys (PROs) via email, phone call, or in-person visit. A total of 111 patients returned surveys via one of these three methods. The remaining 239 patients were contacted over 10 times for follow-up, but ultimately, these patients did not complete the surveys. This is demonstrated in Figure 1.

The PROs were calculated using OrthoToolKit (http://orthotoolkit.com/oswestry/) at the time the patient filled out the surveys, and the answers to each question were recorded separately. Based on the scoring systems used to calculate each PRO, each answer was awarded a specific number of points. These assigned points are pointed out and explained when referenced in each table.

Statistical analysis

The Kruskal-Wallis H test was used to compare the three femoral tunnel drilling techniques (transtibial, anterior medial portal, and anterior portal flexible reaming). This is a rank-based nonparametric test that can be used to find statistically significant differences between two or more groups of an independent variable (in this case, femoral tunnel drilling technique) on the dependent variable in question. The dependent variable can either be continuous or ordinal, all the dependent variables commented within fit this criterion. The distributions of scores for every dependent variable of each femoral tunnel drilling technique demonstrated the same shape. When a significant difference was found via the Kruskal-Wallis H test, a post-hoc test for pairwise comparisons was done to elucidate which groups had significant differences between them. For dichotomous variables, the three femoral tunnel drilling techniques were compared using the chi-square test of homogeneity with the post-hoc test (z-test of two proportions). In addition, to decrease the risk of making a type 1 error, a Bonferroni correction was made for the p-values of each statistical test conducted. All statistical tests were deemed appropriate with the use of Laerd Statistics [19] and conducted using SPSS (IBM SPSS Statistics for Windows, version 28.0; IBM, Armonk, NY, USA).

## Results

A total of 111 patients completed follow-ups at least five years after ACL reconstruction and were included in the study. The average follow-up for the cohort was 6.38 ± 0.99 years. Table 1 reports the median age, race, body mass index (BMI), education level, health insurance type, and smoking status for each technique. No significant differences were found between TT, AMP-RR, and AMP-FR in terms of patient demographics (p-values listed in Table 1).

**Table 1:** Patient demographics based on the femoral tunnel drilling technique. Kruskal-Wallis-H test was used to detect differences between femoral tunnel drilling techniques for age, race, BMI, education, health insurance, and smoking status. AMP-RR: anteromedial portal with rigid reaming, AMP-FR: anteromedial portal with flexible reaming, BMI: body mass index. Answer choice 1.0 for race: White; answer choice 7.0 for education: associate degree; answer choice 8.0 for education: bachelor’s degree; answer choice 4.0 for health insurance: employer-sponsored.

	Transtibial		AMP-RR		AMP-FR		p-value
	Median	Mean Rank	Median	Mean Rank	Median	Mean Rank	
Age (years)	34.0	60.9	36.0	64.2	27.0	45.1	0.051
Race	1.0	53.5	1.0	52.5	1.0	58.4	0.313
BMI (kg/m^2^)	26.5	55.0	26.6	56.0	26.3	53.0	0.924
Education	7.0	51.8	8.0	62.9	7.0	53.1	0.312
Health insurance	4.0	51.1	4.0	61.1	4.0	52.1	0.198
Smoking status	3.0	54.4	3.0	54.3	3.0	56.2	0.887

Table 2 includes the median postoperative PROs and their associated mean ranks for the TT, AMP-RR, and AMP-FR techniques. No significant differences were found between the femoral tunnel drilling techniques on the patients’ outcomes.

**Table 2:** Postoperative patient-reported outcome measurements compared between the femoral tunnel drilling techniques. IKDS: International Knee Documentation Committee subjective evaluation form, KOOS: Knee Injury and Osteoarthritis Outcome Score, RTS: Return to Sport, AMP-RR: anteromedial portal with rigid reaming, AMP-FR: anteromedial portal with flexible reaming

	Transtibial		AMP-RR		AMP-FR		p-value
	Median	Mean Rank	Median	Mean Rank	Median	Mean Rank	
IKDC score	66.5	52.1	75.0	63.8	67.5	55.0	0.319
KOOS total score	84.2	54.4	89.6	62.1	84.9	53.7	0.527
Symptom & Stiffness sub-score (KOOS)	82.0	54.6	85.0	61.0	82.0	54.2	0.649
Pain sub-score (KOOS)	89.0	54.5	93.0	62.5	90.0	53.3	0.470
Function of Daily Living sub-score (KOOS)	94.0	52.5	100.0	62.5	99.5	55.5	0.416
Function in Sports & Recreation sub-score (KOOS)	80.0	56.7	80.0	60.1	75.0	52.5	0.614
Quality of Life sub-score (KOOS)	76.0	55.3	76.0	61.4	73.0	53.1	0.577
RTS after injury total score	57.7	54.3	72.2	58.5	59.8	56.2	0.863
Marx total score	4.0	52.6	7.0	65.0	4.5	53.7	0.236
Pain	2.0	58.4	1.0	49.1	2.0	58.1	0.396
Satisfaction	4.0	55.7	4.0	54.5	4.0	57.3	0.933
Months to RTS after surgery	7.5	39.8	8.5	39.2	8.0	40.9	0.962

Table 3 reports the change in PROs from pre-ACLR to post-ACLR compared between the three techniques. No significant differences were found except in the patient-reported satisfaction (p = 0.018).

**Table 3:** Change in patient-reported outcomes from pre-surgery to post-surgery compared between the femoral tunnel drilling techniques. IKDS: International Knee Documentation Committee subjective evaluation form, KOOS: Knee Injury and Osteoarthritis Outcome Score, AMP-RR: anteromedial portal with rigid reaming, AMP-FR: anteromedial portal with flexible reaming

	Transtibial		AMP-RR		AMP-FR		p-value
	Median	Mean Rank	Median	Mean Rank	Median	Mean Rank	
Δ IKDC score	30.0	51.6	29.0	49.9	30.0	55.1	0.781
Δ KOOS total score	30.3	54.5	22.8	47.1	33.8	53.9	0.586
Δ Symptom & Stiffness sub-score (KOOS)	19.0	49.3	14.0	50.9	23.0	56.9	0.511
Δ Pain sub-score (KOOS)	15.0	51.2	15.0	46.2	27.0	57.9	0.299
Δ Function of Daily Living sub-score (KOOS)	21.0	52.8	18.0	47.9	27.0	55.1	0.645
Δ Function in Sports & Recreation sub-score (KOOS)	50.0	57.4	40.0	51.7	35.0	47.9	0.372
Δ Quality of Life sub-score (KOOS)	46.0	56.3	43.0	47.7	45.0	51.7	0.527
Δ Marx total score	-4.0	54.2	-3.0	55.9	-4.5	47.2	0.444
Δ Pain	-1.0	54.6	-1.0	48.6	-1.0	52.9	0.714
Δ Satisfaction	2.5	61.3	2.0	40.0	2.0	51.5	0.018*

To further elucidate where the significant difference lay, a post-hoc test for pairwise comparison was conducted, shown in Table 4. This found that there was a significant difference between TT and AMP-RR in the change of satisfaction from pre-surgery to post-surgery (p = 0.015), with TT having a larger change in satisfaction. There was no significant difference between TT and AMP-FR or AMP-RR and AMP-FR (Table 4).

**Table 4:** Post-hoc test for the pairwise comparison of patient improvement in satisfaction from pre-surgery to post-surgery for results in Table 3. The significance seen between the three femoral drilling techniques (noted in Table 3) was due to the significant difference found between transtibial (TT) and anterior medial portal rigid reaming (AMP-RR) techniques. Bonferroni correction was applied to all p-values to reduce the risk of making a type 1 error.

	Techniques compared	Significant difference	p-value
Δ Satisfaction	TT vs. AMP-RR	Yes, TT had a larger improvement in satisfaction than AMP-RR.	0.015*
	TT vs. AMP-FR	None	0.424
	AMP-RR vs. AMP-FR	None	0.396

Table 5 reports the change from pre-surgery to post-surgery of PRO individual questions where significant differences were found between groups.

**Table 5:** Changes in specific functions measured in IKDC and KOOS compared between femoral tunnel drilling techniques. Answer choice (points assigned) for IKDC: unable to perform (0), light activities (1), moderate activities (2), strenuous activities (3), very strenuous activities (4). Answer choice (points assigned) for KOOS Q9e: none (0), mild (3), moderate (6), severe (8), extreme (11). IKDS: International Knee Documentation Committee subjective evaluation form, KOOS: Knee Injury and Osteoarthritis Outcome Score, AMP-RR: anteromedial portal with rigid reaming, AMP-FR: anteromedial portal with flexible reaming

	Transtibial		AMP-RR		AMP-FR		p-value
	Median	Mean Rank	Median	Mean Rank	Median	Mean Rank	
Δ IKDC Question 4: During the past 4 weeks, or since your injury, how stiff or swollen was your knee?	0.0	43.6	1.0	54.7	2.0	60.2	0.037*
Δ IKDC Question 9_1: How does your knee affect your ability to go up the stairs?	1.5	60.9	1.0	42.6	1.0	50.2	0.041*
Δ IKDC Question 9_2: How does your knee affect your ability to go down the stairs?	2.0	57.9	0.0	36.7	2.0	57.1	0.008*
Δ KOOS Question 9e: What amount of knee pain have you experienced in the last week while going up or down the stairs?	2.5	55.4	0.0	39.5	3.0	57.9	0.039*

To further elucidate where the significant difference lay, a post-hoc test for pairwise comparison was conducted, as shown in Table 6. A significant difference was found between TT and AMP-FR in the improvement of knee stiffness or swelling (p = 0.047), with AMP-FR having a larger improvement than TT. The post-hoc pairwise comparison between the femoral tunnel drilling techniques and their effect on a patient’s ability to go up the stairs and down the stairs showed that there was a significant difference between TT and AMP-RR (p = 0.043 and p = 0.014), respectively, with TT having a greater positive effect on the patient for both activities. No significant difference was found between AMP-RR and AMP-FR in the ability to go up the stairs. However, there was a significant difference between AMP-RR and AMP-FR on going down the stairs (p = 0.020), with AMP-FR showing greater improvement. No differences were found in the ability to go up or down the stairs between TT and AMP-FR. Finally, a significant difference was found between AMP-RR and AMP-FR in the improvement of knee pain when going down the stairs (p = 0.047) (Table 6).

**Table 6:** Post-hoc test for the pairwise comparison of patient improvement in satisfaction from pre-surgery to post-surgery for the results in Table 5. The significance seen between the three femoral drilling techniques (noted in Table 5) was due to the significant difference found between transtibial (TT) and anterior medial portal rigid reaming (AMP-RR) techniques. Bonferonni correction was applied to all p-values to reduce the risk of making a Type 1 error. IKDS: International Knee Documentation Committee subjective evaluation form, KOOS: Knee Injury and Osteoarthritis Outcome Score, AMP-FR: anteromedial portal with flexible reaming

	Techniques compared	Significant difference	p-value
Δ IKDC Question 4: During the past four weeks, or since your injury, how stiff or swollen was your knee?	TT vs. AMP-RR	None	0.423
	TT vs. AMP-FR	Yes, AMP-FR had a larger improvement from pre-surgery to post-surgery than TT.	0.037*
	AMP-RR vs. AMP-FR	None	1.000
Δ IKDC Question 9_1: How does your knee affect your ability to go up the stairs?	TT vs. AMP-RR	Yes, TT had a larger improvement from pre-surgery to post-surgery than AMP-RR.	0.043*
	TT vs. AMP-FR	None	0.311
	AMP-RR vs. AMP-FR	None	0.942
Δ IKDC Question 9_2: How does your knee affect your ability to go down the stairs?	TT vs. AMP-RR	Yes, TT had a larger improvement from pre-surgery to post-surgery than AMP-RR.	0.014*
	TT vs. AMP-FR	None	1.000
	AMP-RR vs. AMP-FR	Yes, AMP-FR had a larger improvement from pre-surgery to post-surgery than AMP-RR.	0.020*
Δ KOOS Question 9e: What amount of knee pain have you experienced in the last week while going up or down the stairs?	TT vs. AMP-RR	None	0.106
	TT vs. AMP-FR	None	1.000
	AMP-RR vs. AMP-FR	Yes, AMP-FR had a larger improvement from pre-surgery to post-surgery than AMP-RR.	0.047*

Table 7 lists the number of patients who were able to return to sport (since surgery, at pre-injury level, regardless of level), felt that it was important that they return to their sport, had additional procedures on the affected knee (for meniscus tears or cyclops lesions), and required revision ACL surgery. No significant differences were found between the three femoral tunnel drilling techniques.

**Table 7:** Return to sport and additional procedures after ACL reconstruction compared between the femoral tunnel drilling techniques. The chi-square test of homogeneity with the post-hoc test (z-test of two proportions) found no significant differences for pairwise comparison when analyzing the above outcome measures. ACL: anterior cruciate ligament, RTS: return to sports, ACLR: anterior cruciate ligament reconstruction

	Transtibial		Anterior medial portal		Anterior portal flexible reaming		p-value
	N	%	N	%	N	%	
RTS since ACL surgery	23	52.3	18	66.7	20	50.0	0.495
Important for patient to RTS	25	56.8	20	74.1	30	75.0	0.337
RTS at pre-injury level	20	45.5	17	63.0	21	52.5	0.357
RTS regardless of level	30	68.2	20	74.1	26	65.0	0.734
Additional procedures on affected knee since primary ACL surgery	7	15.9	4	14.8	7	17.5	0.956
Required revision ACL surgery	0	0.0	2	7.4	2	5.0	0.224
Required meniscus repair/debridement or meniscectomy post-ACLR	1	2.3	3	11.1	4	10.0	0.261
Required excision of cyclops lesion post-ACLR	1	2.3	0	0.0	0	0.0	0.464
Total number of patients	44		27		40		

In summary, no differences were found between the three groups in terms of demographics or postoperative PROs (IKDC, KOOS, KOOS sub-scores, RTS post-injury, Marx score, pain, satisfaction, and months to RTS). However, the change between pre-surgery PROs and post-surgery PROs did show some differences. TT, when compared to AMP-RR, had a greater increase in satisfaction and greater improvement in a patient’s ability to go up and down the stairs from pre-surgery to post-surgery. AMP-FR, when compared to TT, had greater improvement in the patient’s knee stiffness/swelling. AMP-FR, when compared to AMP-RR, had greater improvement in knee pain and the ability to go down the stairs. No differences in return to sport, additional procedures on the affected knee, or revision surgery rates were found.

## Discussion

The importance of femoral tunnel drilling in ACLR is clear. A 2012 study by the MARS group showed that failure of primary ACLRs is due, at least in part, to malpositioned femoral tunnels [20]. In a 2020 systematic review of over 3,000 patients, it was found that 63% of technical failures of ACLR are due to poor positioning of the femoral tunnel [21]. Postoperative sagittal and rotational control of the knee is impacted by the femoral tunnel [1]. With this amount of impact on patient outcomes, such as ACLR failure, specific attention must be focused on the techniques available to orthopedic surgeons.

This study focuses on three femoral tunnel drilling techniques (TT, AMP-RR, and AMP-FR). Many previous studies aim to compare two techniques (most often TT and anteromedial portal) in a cohort of patients, but most do not separate out anterior medial portal rigid reaming versus the flexible reaming as is done in this study. More specifically, the current literature focuses on the total score of PROs, whereas here, we do a deep dive into the individual questions that affect the overall PRO score to elucidate impacts on specific activities, functions, or symptoms. These differences in individual activities, potentially due to the femoral tunnel drilling technique used, could be masked by the overall PRO score not being statistically significantly different.

Other studies have shown conflicting results when comparing the anteromedial portal and TT methods. Metso et al. compared 60 patients with anterior medial portal to 58 patients with TT methods used and found no significant differences in outcomes at the two-year follow-up mark [7]. Rates of revision ACLR and outcomes scores (Lysholm score, Tegner activity score, and pivot shift test) did not differ between the use of anterior medial portal and TT techniques in a New Zealand ACL registry comparing femoral tunnel drilling techniques of studies with follow-up of one year [9] or at two years [22]. A meta-analysis reported that anteromedial portal drilling had significantly improved outcome scores as compared to outside-in and TT methods for double-bundle ACLR with follow-up ranging from two days to three years [23]. A prospective study comparing TT to the anteromedial portal method showed higher odds for a repeat ipsilateral knee surgery in the TT technique relative to patients with the anteromedial portal technique [24]. Our study did not find a difference between the femoral tunnel drilling techniques in terms of ipsilateral knee reoperations. In addition, when reoperations were broken up by their reason, no differences were seen in reoperation rates for revision ACL, meniscal tears, or cyclops lesions. By contrast, a 19,000-patient retrospective study of primary ACLR concluded that TI techniques (anteromedial portal, outside-in, and retrodrilling methods) carried a higher risk for aseptic revision compared to the TT technique [10]. The conclusions in the literature comparing anteromedial portal and TT, when looked at as a whole, are confusing; articles have reported TT being inferior to anteromedial portal, anteromedial portal being inferior to TT, or no difference between the two on patient outcomes. Notably, none of these studies specified whether rigid or flexible reaming was used in the anteromedial portal technique. This could have large implications on the results, as AMP-FR is thought to have a decreased risk for posterior wall blowout and damage to lateral structures as compared to AMP-RR [1,3,5,6,8]. If grouped together, results could be inaccurate.

One study that did differentiate between anterior medial portal rigid reaming and flexible reaming when compared to TT was a case series published by Shah et al. Although the authors focused on similar outcomes, they had a smaller cohort of patients (51 patients) and a shorter follow-up period of two years [25], whereas we report on patients with over a six-year mean follow-up. Like the results presented in this study, they did not find postoperative differences between the femoral tunnel drilling techniques on IKDC, KOOS, or Marx scores. However, we did find a significant difference in the improvement of patient satisfaction, with TT having a larger improvement than AMP-RR but no differences between other pairs of techniques.

Another potential reason, aside from the grouping of AMP-RR and AMP-FR, that studies have conflicting conclusions on femoral tunnel drilling techniques is that there are underlying significant differences in their effects on specific activities/symptoms. To uncover these factors, our deep dive into the cohort’s outcome scores revealed that significant differences lay in the ability to climb up and down the stairs and knee stiffness/swelling. The results suggest that AMP-RR may be inferior to AMP-FR and TT in terms of stair climbing at long-term follow-up after ACLR. It could be argued that AMP-FR may be superior to both TT and AMP-RR, as AMP-FR was found to have at least equal or greater improvement in both overall PRO scores and individual PRO questions. Moreover, AMP-FR showed greater improvement in a patient’s knee stiffness and swelling than TT. In theory, this makes sense as flexible reaming has been shown to have the benefits of AMP-RR (closer positioning to the anatomic ACL footprint) with less risk of the disadvantages (posterior wall blowout and damage to lateral knee structures).

A retrospective study of 284 patients compared AMP-FR to AMP-RR at six months post-surgery, finding no significant difference in graft failure, IKDC scores, KOOS scores, or functional tests. However, this is a short-term comparison between rigid and flexible reaming through an anteromedial portal [8], whereas we report on long-term results reflecting the durability of the reconstruction when using rigid or flexible reaming. The results presented here suggest that AMP-FR does not have superior results to AMP-RR when comparing overall PRO scores but does show superiority when analyzing improvement in ability to and pain when going down the stairs.

These differences in stair climbing ability, stiffness, and swelling could influence a patient’s postoperative satisfaction and may be attributed to the difference seen between the anteromedial portal and TT in other studies. It is known through both biomechanical and clinical studies that the anteromedial portal provides better results for ACLR when quantified by pivot-shift tests and IKDC scores, as it attempts to mirror the anatomical ACL placement. Around 55% of surgeons prefer to use the anteromedial portal (whether with rigid or flexible reaming), 32% prefer retrograde drilling (not assessed in this study), and 14% prefer the transtibial technique [26]. A definitive statement on which femoral tunnel drilling technique is indisputably superior cannot be made yet however as further research, ideally randomized controlled trials, is needed to settle the discrepancies seen throughout the current literature on this topic.

In addition, this study suggests that one technique may not be clearly superior/inferior as compared to the other techniques when it comes to overall PROs. However, when taking a deep dive into the individual questions on PRO surveys, differences can be found that would otherwise be masked by statistically insignificant findings on the overall PRO scores. It is important to recognize these differences between TT, AMP-RR, and AMP-FR in the improvement of stair climbing and swelling/stiffness as these likely directly affect a patient’s change in satisfaction from pre-ACLR to post-ACLR.

There are several limitations to note in this study. First, despite efforts to contact all eligible patients for follow-up, a substantial number did not complete the surveys. This attrition could introduce bias if patients who lost to follow-up had different outcomes than those who responded. Second, although the study aimed to compare three specific femoral tunnel drilling techniques (transtibial, anteromedial portal with rigid reaming, and anteromedial portal with flexible reaming), there may have been variability between each technique group as not all four orthopedic surgeons performed all three femoral tunnel drilling techniques; each had their preferred method. Although the training level, practice setting, and years of experience of a surgeon are known to predict which femoral tunnel drilling technique is used [26], it is likely that each surgeon chose the technique at which they could perform at the highest technical level. Surgeon experience and skill level could influence outcomes but were not accounted for in the analysis. Finally, our study focused on patient-reported outcome scores (PROs) rather than objective measures, such as knee laxity testing, graft stability assessments, or radiographic evaluations of tunnel position, which were not our primary focus. These objective measures could provide a more comprehensive evaluation of the effectiveness of each technique and should be considered in future studies.

## Conclusions

When comparing overall postoperative PROs (IKDC, KOOS, RTS post-injury, Marx, pain, and satisfaction scores) and the need for reoperation in patients treated with TT, AMP-RR, and AMP-FR femoral tunnel drilling techniques in ACLR, no technique was found to be clearly superior or inferior to the others. However, TT was found to have a greater improvement in patient satisfaction and in a patient’s ability to go up and down the stairs from pre-surgery to post-surgery. AMP-FR, when compared to TT, had greater improvement in the patient’s knee stiffness/swelling. AMP-FR, when compared to AMP-RR, had greater improvement in knee pain and the ability to go down the stairs. Additional prospective studies, particularly randomized controlled trials, are needed to further investigate these functional improvements.
